# Supplementary material for: Microbial Enrichment Techniques on Syngas and CO2 Targeting Production of Higher Acids and Alcohols
Source: Molecules. 2023 Mar 11;28(6):2562. doi: 10.3390/molecules28062562 (PMC10056454; doi:10.3390/molecules28062562)
Supplement: Supplementary file 1 [file molecules-28-02562-s001.zip › molecules-2236637-supplementary.pdf]

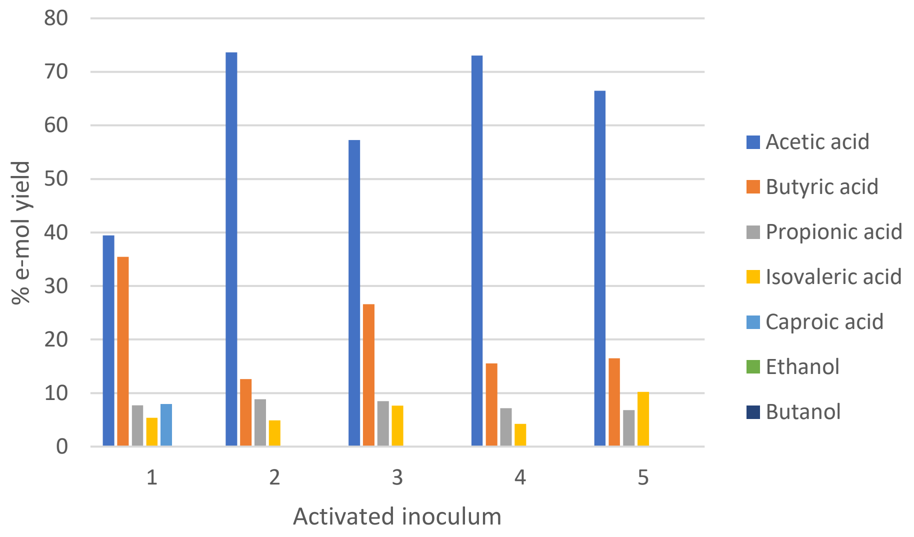

Figure S1. Distribution of % e-mol yields of all products at the end of the activation of the inoculum.
